# The effects of storage and hop extract on aroma and flavour compounds in Balkan-style sausages packed under a CO_2_-containing anaerobic atmosphere

**DOI:** 10.1016/j.heliyon.2020.e05251

**Published:** 2020-10-14

**Authors:** Diego E. Carballo, Sonia Andrés, Francisco Javier Giráldez, Ali Khanjari, Irma Caro, Diego Llamazares, Sabina Operta, Javier Mateo

**Affiliations:** a Department of Hygiene and Food Technology, Faculty of Veterinary Medicine, University of León, León, 24071, Spain; b Instituto de Ganadería de Montaña, CSIC-Universidad de León, Finca Marzanas s/n, Grulleros, León, 24346, Spain; c Department of Food Hygiene, Faculty of Veterinary Medicine, University of Tehran, P.O. Box 14155-6453, Tehran, Iran; d Department of Nutrition and Food Science, Faculty of Medicine, University of Valladolid, Valladolid, 47005, Spain; e Faculty of Agricultural and Food Science University of Sarajevo, Institute of Food Science and Technology, Zmaja od Bosne 8, Sarajevo, 71000, Bosnia and Herzegovina

**Keywords:** Food science, Food analysis, Microbiology, Natural antimicrobial, Natural antioxidant, Modified atmosphere packaging, Volatile compounds, Microbial spoilage

## Abstract

Changes in the levels of pH, lactic acid, acetic acid, headspace volatile compounds, and spoilage odour intensity during a 35 day refrigerated storage period in two sets of a Balkan-style fresh sausage, a control sausage (CON) and a sausage containing an aqueous hop extract (HOP), packaged under a 20% CO_2_ and 80% N_2_ atmosphere were evaluated. Storage resulted in progressive sausage acidification and increased the levels of acetic acid, 1-methylbutanol, ethyl acetate, ethyl hexanoate, and 2-ethylhexanol; all of which are associated with anaerobic microbial metabolism under sugar restricted conditions. Storage decreased the levels of hexanal, heptanal, pentanol, and garlic-derived organosulfur compounds. Hop extract showed oxygen scavenging abilities, and decreased the levels of the volatile compounds derived from lipid auto-oxidation while contributing to the presence of specific terpene compounds. The use of hop extract did not improve the shelf life of sausages packed under anaerobic atmosphere. The spoilage odour appeared in both types of sausages on the 14th day, and it was considered strong from day 21 onwards.

## 1 Introduction

Fresh sausages are commonly made from minced meat to which salt and different spices and condiments are added. These sausages are highly perishable. In order to extend their shelf life, low storage temperatures combined with packaging under CO_2_-containing anaerobic atmospheres are utilised (Cocolin et al., 2004; Hugo and Hugo, 2015). In fresh sausages stored under these conditions, lactic acid bacteria (LAB), mainly *Lactobacillus* species, together with *Brochothrix thermosphacta* and different *Enterobacteriaceae* species become the predominant microbiota and the principal spoilage organisms (Benson et al., 2014). The growth of these microorganisms has been monitored in different studies and associated with a negative effect on the sausage flavour, resulting in a shelf life of 10–20 days (Casaburi et al., 2015; Fougy et al., 2016; Martínez et al., 2005; Pothakos et al., 2015; Ruiz-Capillas and Jiménez-Colmenero, 2010; Salinas et al., 2014). The volatile compounds associated with the spoilage of meat packed under anaerobic atmosphere have been extensively described (Casaburi et al., 2015). However, to the best of our knowledge, no studies on storage-related changes in the flavour compounds of fresh sausages are available, with the exception of that by Rux et al. (2019). These authors reported volatile compounds originated from spices, meat, and microbial activity, and suggested that further investigation was needed to elucidate the role of microorganisms in the generation of volatile compounds.

Antimicrobial additives are frequently used to increase the shelf life of fresh sausage and, due to the trend of replacing synthetic antimicrobials, the use of natural bioactive compounds is increasingly being considered (Hugo and Hugo, 2015). Considering their high content of antimicrobials, which are especially effective against Gram-positive bacteria (Hrnčič et al., 2019), hop extracts, commonly used in brewing, seem to be an appropriate natural antimicrobial agent for use in the meat industry (Singh et al., 2014). No studies where hop extracts have been added to fresh sausages to improve their shelf life have been found in the literature.

Therefore, the aims of this study were to describe and gain knowledge of the changes in flavour compounds in the headspace of Balkan-style fresh lamb sausages (CON treatment) during refrigerated storage under a CO_2_-containing anaerobic atmosphere and to assess the effect of using hop extract (HOP treatment) as a natural antimicrobial and antioxidant ingredient against those changes. This is a continuation of a previous study (Carballo et al., 2019) which evaluated the changes in microbial populations and biogenic amine formation due to storage time and the use of hops.

## 2 Materials and methods

### 2.1 Sausage preparation and treatments

The sausages used in the study were the same as those previously described by Carballo et al. (2019). The right-hand leg meat from six male Assaf lambs (49.5 ± 2 kg body weight) reared under a conventional intensive feeding system was used in this research. All handling practices were approved by the Spanish Research Council (CSIC) Animal Experimentation Committee (protocol number 100102/2017-4).

The legs were separated from the carcasses and deboned 24 h post-mortem. The leg meat was cut into approximately 3 cm cubes and trimmed of visible fat. Lean meat from each leg was vacuum-packaged and frozen (−20 °C) until further use (no more than three months). An aqueous hop extract was prepared using recently cropped Nugget-variety hops that were kindly provided by a local producer (Orbigo Valley S.L., Madrid, Spain). It contained α-acids, β-acids, and co-humulone at levels of 4.8–5.3%, 12–16%, and 22–28%, respectively. In brief, 50 g of hops were boiled in water for 30 min, and the mixture was filtered through a Whatman no. 1 filter paper (GE Healthcare Europe GmbH, Barcelona, Spain). The volume of the filtrate was made up to 1 l with water, and the hop extract was frozen (−18 °C) until further use. The antioxidant potential of the hop extract used in the experiment was 0.004 mmol Trolox per ml of extract as evaluated by 2,2-diphenyl-1-picrylhydrazyl analysis (Serpen et al., 2012).

Three batches of lamb sausages were produced on different days at the Faculty of Veterinary Medicine, University of León (Spain). The sausage preparation was based on a Balkan region recipe (ćevapi sausages): minced lean lamb, 950 g (two lamb legs were used per batch); common salt, 20 g; sodium bicarbonate, 3 g; spice infusion (filtered solution obtained by boiling garlic and black pepper in water), 20 ml; water, 30 ml. Two sets of sausages were prepared per batch: a control sausage (CON), following the recipe; and a hop sausage (HOP), where the same volume of hop extract replaced the water (30 ml). This amount of hop extract (30 ml of extract per kg of sausage mix) contained the water-extractable compounds from 1.5 g of hops, which is a comparable amount to that commonly used per litre of wort in brewing, i.e. 1-2 g hops/l. Furthermore, this amount did not negatively affect the Balkan-style sausage flavour, i.e. unpleasant bitterness, according to the results of preliminary taste studies carried out with consumers at the University of Sarajevo.

The day before the sausage preparation, the leg meat and hop extract were thawed at 5 °C. The sausage-making process consisted of an initial step of mincing the meat using a butcher's mincer (5 mm diameter sieve) and mixing the meat with common salt for 10 min. The mixture was then kept covered with cling film at 4 °C for 24 h. Next, the spice infusion, sodium bicarbonate, and water (CON) or hop extract (HOP) were added to the salted minced meat, which was mixed for 5 min. The sausage mixtures were then stuffed into lamb casings (20/22 cm diameter), and the sausages were air-dried for 3 h at 12 °C and then cut into 100 g portions. The portions were individually packed into plastic film bags [12 × 20 cm; 150 μm thickness; oxygen permeability of 30 cm^3^/(m^2^ bar 24 h) at 23 °C and 0% relative humidity] under a CO_2_ and N_2_ (20% and 80%) atmosphere at 7500 kPa. Finally, the packed sausage portions were stored in darkness at 2 ± 1 °C for up to 35 days.

### 2.2 Sausage sampling

One portion of each of CON and HOP raw sausage was sampled at days 0 (day of packaging), 7, 14, 21, 28, and 35 of storage for microbial and chemical analysis (carried out in duplicate), i.e. total aerobic mesophilic counts, pH, lactic and acetic acids contents, and volatile compound contents.

Total aerobic mesophilic counts and pH analysis were carried out just after sampling; the remaining sausage sample was frozen at −30 °C for up to 3 months and then thawed (24 h, 4 °C) before analysis. Another sausage portion of CON and HOP raw sausages were sampled on each storage day and frozen at −18 °C for evaluation of spoilage odour. Prior to analysis, the CO_2_ and O_2_ concentrations inside the packaging bags were analysed on days 7–28. Due to technical reasons, it was not possible to obtain the measurements at day 35, and we assumed that at day 0 the concentrations were that of the gas cylinder (20% of CO_2_ and 80% of N_2_).

### 2.3 Microbiological analysis

For the aerobic mesophilic counts (AMC), 25 g (±0.1 g) of raw sausage samples were homogenised for 2 min with 225 ml of 0.1% peptone water and 0.85% NaCl in a Stomacher-400 circulator (Seward, West Sussex, UK). Serial decimal dilutions were then prepared, and 1 ml aliquots of the appropriate dilutions were plated out onto Standard Plate Count Agar (PCA; Oxoid Ltd, Basingstoke, UK). Finally, the cultured plates were incubated at 30 °C for 48 h.

### 2.4 Instrumental chemical analysis

The CO_2_ and O_2_ composition of the modified atmosphere in the packaging bags was analysed using OXIBABY equipment (Cambridge Sensotec, St Ives, UK). The pH was determined using a BasiC 20 pH meter (Crison Instruments, Barcelona, Spain) equipped with a 52–32 pH penetration electrode.

Lactic and acetic acid contents in the sausage samples were extracted by homogenising 10 g of sausage in 40 ml of 4.5 mM H_2_SO_4_ using a T10 basic Ultraturrax (IKA-Werke, Staifen, Germany), with further filtration through Whatman no. 54 filter papers (GE HealthCare, Little Chalfont, United Kingdom). The concentrations were determined following the method described by Bruna et al. (2003). A high-pressure liquid chromatograph (Model 2690; Waters Corporation, Milford, MA, EEUU equipped with a 300 mm × 7.8 mm Aminex HP-87H ion-exchange column (Bio-Rad Laboratories, Hercules, CA, USA) and 3 mM H_2_SO_4_ as eluent were used for separation. Detection and quantification were carried out using a diode array detector (Model 996, Waters Corporation) and adequate solutions of lactic and acetic acid standards (Sigma-Aldrich Química, Madrid, Spain) in 4.5 mM H_2_SO_4_.

The extraction of volatile compounds from the sausages was carried out using solid-phase micro-extraction (SPME) fibres (75 μm Carboxen/polydimethylsiloxane-coated fused silica fibre, 1 cm coating length) from 4 g of homogenised sausage sample in 15-ml screw cap vials. Vials with the samples were incubated at 40 °C in a water bath with sonication (Bransonic 221; Branson, Danbury, CT, USA) for 15 min (equilibration) and then an additional 40 min (exposition to the fibre). Extraction was followed by injection (for 2 min at 260 °C in splitless mode) and gas chromatography separation, using a CG 7890A equipment (Agilent Technologies, Santa Clara, FL, USA) equipped with a 60 m × 0.25 mm ID, 0.25 μm film thickness, DB-5MS column (J&W Scientific, Folsom, CA, USA). The oven temperature was programmed at 35 °C (1 min), 35 °C–50 °C (10 °C/min), 50 °C–200 °C (4 °C/min), 200 °C–250 °C (50 °C/min), and 250 °C (11 min). The carrier gas (helium) flow was 1 ml/min. Detection was carried out using an MSD 5975C simple quadrupole mass spectrometry detector (Agilent Technologies) operating in the electron impact mode (70 eV, 50 lA) and scanning from 40 m/z to 350 m/z at 3.94 scans/s.

Identification of volatile compounds was carried out by spectra comparison using the MSD ChemStation software, searching the NIST/EPA/NIH-98 Mass Spectral Database, personal interpretation, and comparison of the linear retention indexes, experimentally calculated using a series of n-alkanes (Hydrocarbons/C5–C20; Sigma-Aldrich, St. Louis, MO, USA), with those from the literature for a DB-5 capillary column or similar (Adams, 2007; Kondjoyan and Berdagué, 1996; Linstrom and Mallard, 2020; Vázquez-Araújo et al., 2013). The methods followed for extraction, identification, and detection were those described by Del Blanco et al. (2017) with a modification in the sample incubation phase, which was carried out in this study using sonication. The peak areas were transformed to ng of hexanal equivalents by comparing them with those of a hexanal external standard curve in hexane. Hexanal was used because it is one of the major volatile compounds in sausages and has an intermediate polarity and chromatography retention time.

### 2.5 Spoilage odour analysis

The odour analysis was carried out at the Instituto de Ciencia y Tecnología de Alimentos (University of León, Spain). It consisted of evaluating the odour intensity associated with spoilage of CON and HOP sausages at the different storage times. Six assessors were selected and trained before evaluation (four males and two females between 25 to 60 years old). The procedure followed for training and the test was based on that described by Martínez et al. (2016). Training consisted of three one-hour open discussion sessions to recognise and memorise the spoilage odour using reference sausages and to establish and practice the testing procedure and scale used. The reference sausages consisted of 100 g portions of the experimental sausages that were frozen on the day they were stuffed into casings (sausage with no perceivable spoilage odour) and after 42 days of refrigerated storage (sausage with extremely perceivable spoilage odour). Reference samples were prepared (for each panellist) by placing 10 g of reference sausage (thawed for 24 h at 4 °C) into tightly closed screw-cap vials (50 ml headspace, 22 mm diameter screw cap) covered with a layer of aluminium foil and tempered at room temperature (22 °C) for no less than an hour.

The analysis was carried out in individual booths under artificial green light to prevent panellists from associating the eventual colour differences in the sausage batter with the day it was stored. There were seven tasting sessions in total. In each session, up to six samples were tasted by at least five out of the six trained panellists; five is the lowest number of panellists recommended for product-oriented sensory analysis (Watts et al., 1989). All panellists smelled the whole sample set in random order. At the beginning of each session, panellists smelled vials containing the above-mentioned reference samples as a reminder. Samples were then analysed one by one as a blind testing. Panellists were given randomly ordered vials with the testing sausage samples prepared as explained for reference samples. They were asked to open the vial, take it with the fingers by the bottom, place the neck of the vial so it almost touched their nose, slowly inhale the air contained in the vial for a few seconds with both nostrils open, and then evaluate the spoilage odour. They were permitted to repeat the inhalation if needed. Spoilage odour was rated using a four-point scale (0 = not perceivable, 1 = slightly perceivable, 2 = considerably perceivable, and 3 = extremely perceivable, as in the reference spoiled sample). Between samples, panellists had to smell a vial with a mixture of odorant spices and herbs to avoid the adaptation of olfactory stimuli, as recommended by Doty (2017).

### 2.6 Statistical analysis

The microbial and chemical characteristics of the CON and HOP sausages were analysed in duplicate on each of the sampling days for each of the three batches produced, and each treatment. The mean value of the duplicates was calculated, and the means were analysed by two-way analysis of variance (ANOVA) with treatment and storage day as fixed factors. When the fixed factors or their interaction showed significant differences (*P* < 0.05), ANOVA was followed by Duncan's multiple range test.

For the spoilage odour analysis, panel reliability was assessed by testing 12 samples in duplicate (six CON and six HOP, one per sampling day and treatment, each duplicate was tested on the same day) and calculating the mean of the errors for each panellist. The errors were calculated as √ [∑ (Sr_1_ − Sr_2_)^2^/2N], where Sr_1_ and Sr_2_ are the scores for replicates 1 and 2, respectively, and N is the number of samples tested in duplicate for each panellist. The mean error (±standard deviation) obtained was 0.6 (±0.3). To calculate the mean scores given to the sausages per day and treatment (as given in Table 3), the median value from the panellist's scores for each sample tested was calculated first, and then the mean value from the medians for each of the three sausage batches was obtained and rounded up to the nearest 0.5.

## 3 Results and discussion

### 3.1 Changes in microbial counts, pH, lactic and acetic acids content, and atmosphere composition

**Figure 1:**
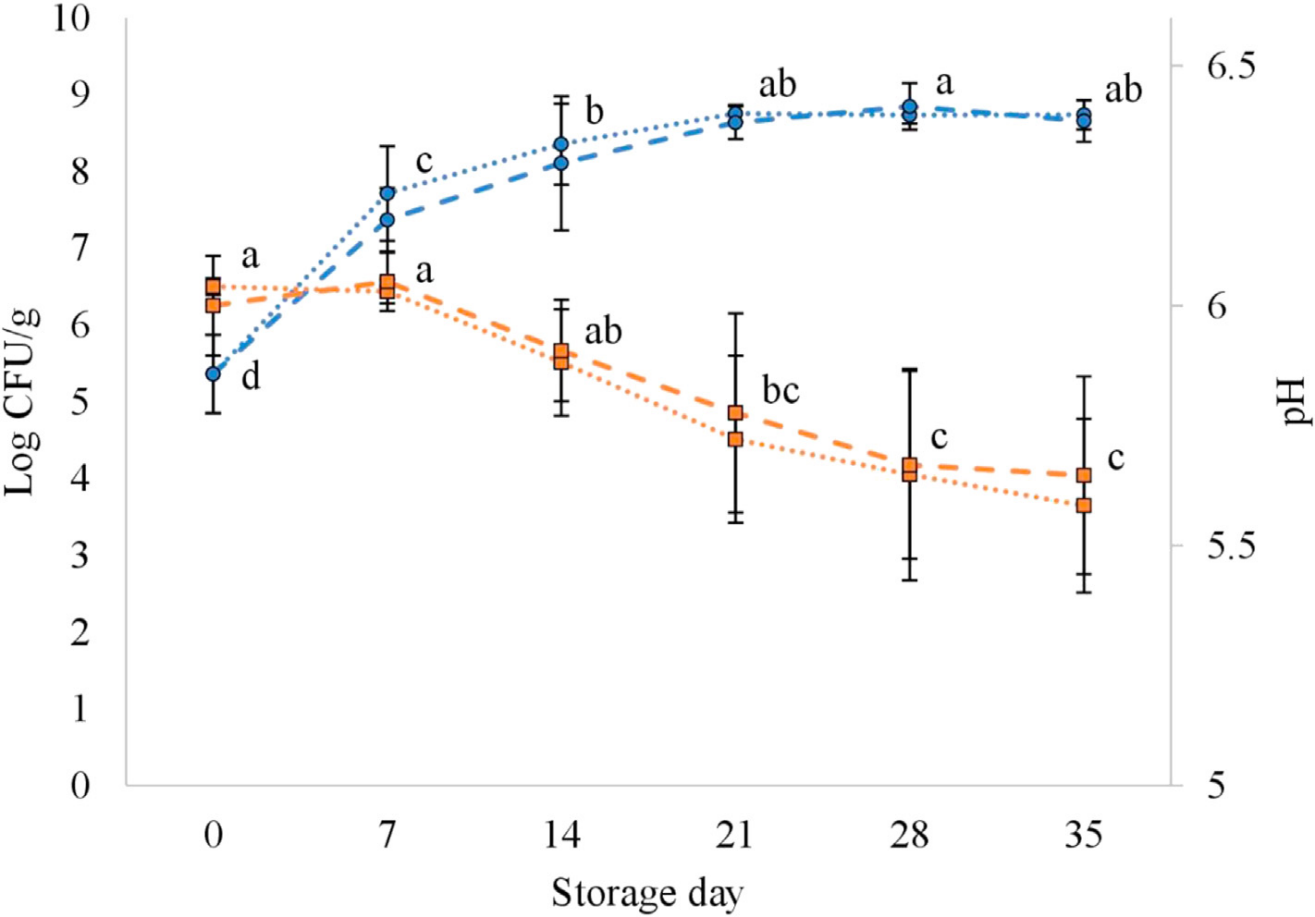
Changes in total viable bacterial counts () and pH () in fresh lamb sausages stored refrigerated (2 °C) under a 20% CO_2_ and 80% N_2_ atmosphere. Dotted lines, control sausages (CON); dashed lines, sausages containing 30 ml/kg of a hop extract (HOP). Vertical bars represent the standard deviation from the mean of each treatment (n = 3). a–d: different superscripts on the storage day values indicate statistical difference (*P* < 0.05; Duncan's multiple range test).

The time-related changes in total aerobic mesophilic counts (AMC) and pH of CON and HOP sausages are depicted in Figure 1. Counts increased sharply during the first week of storage and reached final values of between 8 and 9 Log colony-forming units (CFU) per gram of sausage on day 21 when the stationary growth phase was reached. As seen in a previous study, LAB was the most predominant group by far in the CON and HOP sausages from day seven onwards (Carballo et al., 2019). This study also reported that among LAB species, *Lactobacillus* *sakei* was the principal one. *B. thermosphacta* and facultative anaerobic *Enterobacteriaceae* were also abundant among the sausage microbiota. Similar AMC growth patterns to those of the present study have been described for fresh sausages refrigerated under O_2_ deprivation (Lerasle et al., 2014; Ruiz-Capillas and Jiménez-Colmenero, 2010).

The pH of the sausages decreased steadily (*P* < 0.05) from day 7 to day 28, by approximately 0.3 units. Significant differences from day 0 were found from day 21 onwards. This decrease was due to LAB fermentation of the sugars present in the sausage mix, originating from the meat, garlic, and pepper, and also to dissolution of CO_2_ gas from the packaging into the sausage (Lerasle et al., 2014). The maintenance of pH from day 0–7, despite great microbial growth (and thus fermentation) in this week, could be explained by the bicarbonate–carbonic acid buffer (pK = 6.1), since sodium bicarbonate was used as an ingredient.

**Figure 2:**
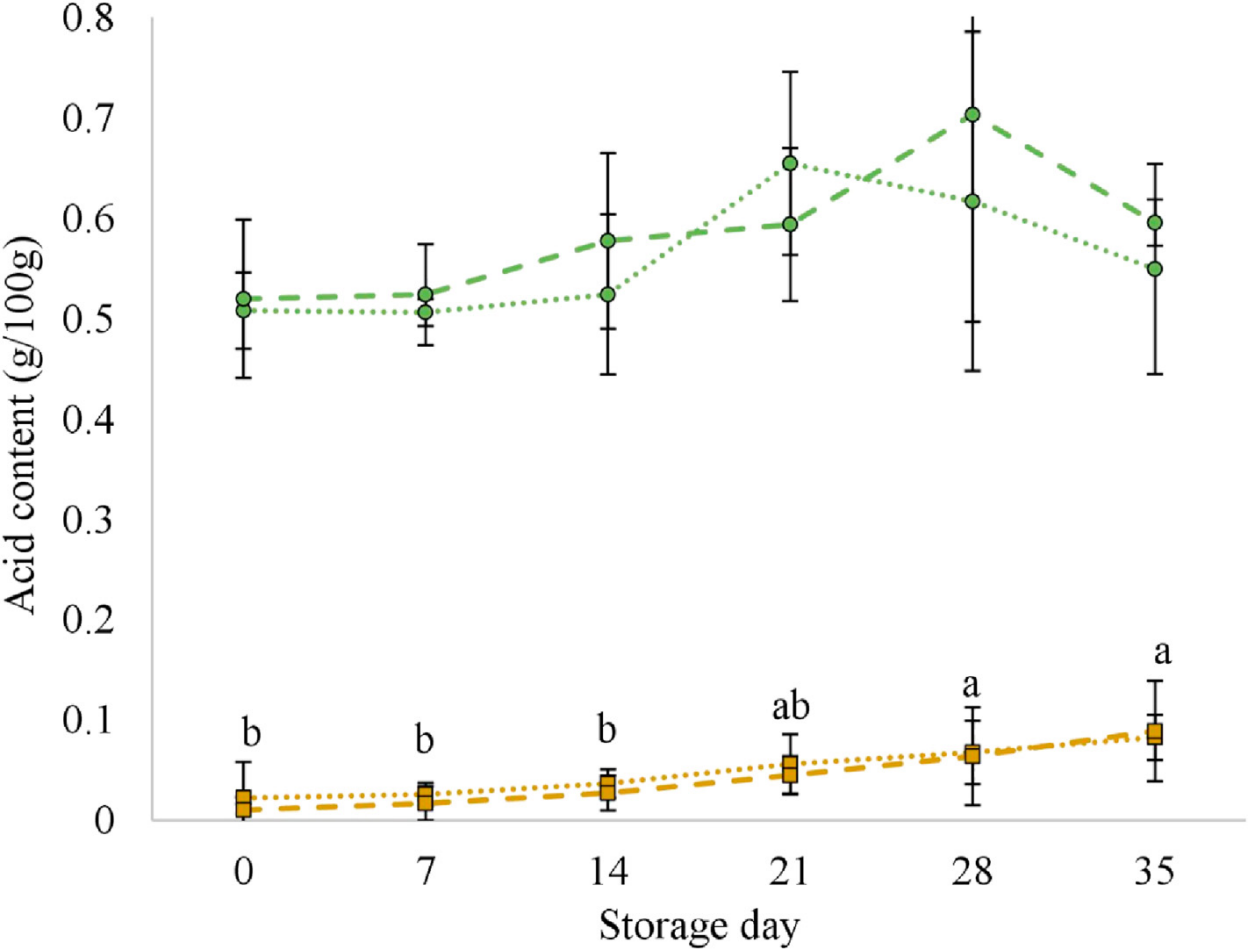
Changes in lactic acid () and acetic acid () content in fresh lamb sausages stored refrigerated (2 °C) under a 20% CO_2_ and 80% N_2_ atmosphere. Dotted lines, control sausages (CON); dashed lines, sausages containing 30 ml/kg of a hop extract (HOP). Vertical bars represent the standard deviation from the mean of each treatment (n = 3). abc: different superscripts for the storage day values indicate statistical difference (*P* < 0.05; Duncan's multiple range test).

The changes in lactic and acetic acids content are shown in Figure 2. No significant changes were observed for lactic acid levels due to storage. The mean concentrations of this acid were always in the range between 0.5 and 0.7 g/100 g. In contrast, acetic acid content increased significantly during storage until reaching amounts of approximately 0.1 g/100 g; significant differences from day 0 were found at days 28 and 35. The use of hop extract had no significant effect on the levels of both acids.

Lactic and acetic acids would have been produced in the sausages as end-products of sugar fermentation by the predominant bacteria, i.e. lactic acid by LAB, *B. thermosphacta*, and *Enterobacteriaceae*, and acetic acid by heterofermentative LAB and *Enterobacteriaceae* (Müller, 2008; Pothakos et al., 2015; Stanborough et al., 2017). The lack of a significant accumulation of lactic acid, despite it being produced via sugar fermentation, could be explained by its simultaneous degradation by lactate-utilising *lactobacilli*, which would result in additional acetic acid production (Gänzle, 2015). The generation of acetic acid could also be the result of the microbial use of pyruvate, which is highly generated from microbial catabolism of amino acids in the absence of sugars. The ability to use pyruvate by *L. sakei*, the most predominant LAB species in the sausages of this study (Carballo et al., 2019), for the production of energy has been reported (Montanari et al., 2018).

**Figure 3:**
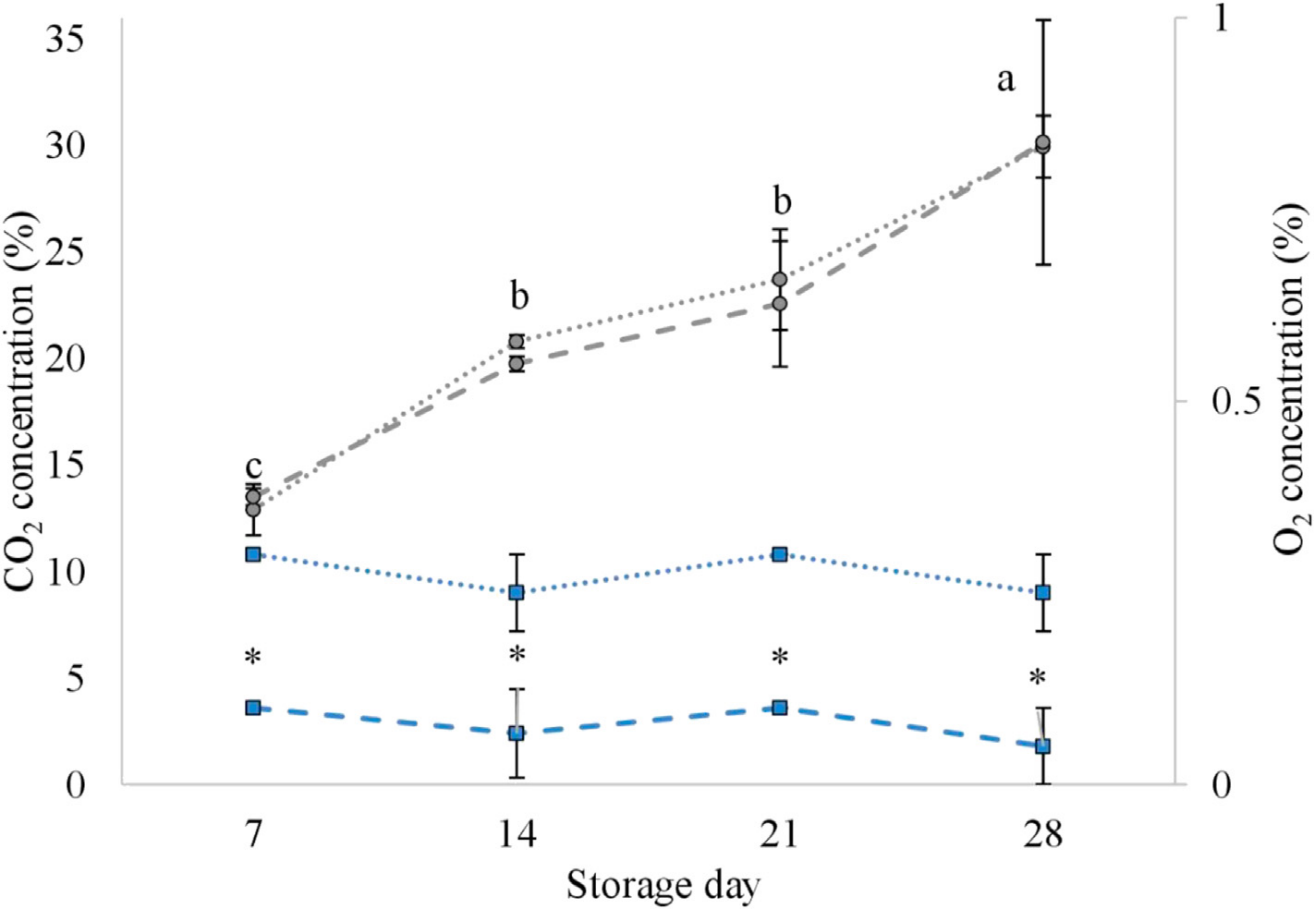
Changes in CO_2_ () and O_2_ () concentrations in the modified atmosphere of fresh lamb sausages stored refrigerated (2 °C) under a 20% CO_2_ and 80% N_2_ atmosphere (initial conditions). Dotted lines, control sausages (CON); dashed lines, sausages containing 30 ml/kg of a hop extract (HOP). Vertical bars represent the standard deviation from the mean of each treatment (n = 3). ∗: indicates the statistical difference in gas concentration between sausage type within a storage day (*P* < 0.05). abc: different superscripts for the storage day values indicate statistical difference (*P* < 0.05; Duncan's multiple range test).

The percentages of CO_2_ and O_2_ in the packaging atmosphere during the storage of CON and HOP sausages are shown in Figure 3. Over time, the amount of CO_2_ increased. It reached levels close to 30% on day 28 of storage, which was accompanied by pack distension. The increase in CO_2_ levels could be directly attributed to bacterial metabolism, i.e. decarboxylation and oxidation reactions of amino acids, sugars, pyruvate, keto-acids (Carroll et al., 2016; Lerasle et al., 2014; Müller, 2008; Xiao and Lu, 2014). Furthermore, in this study, an important contribution to the CO_2_ accumulated in the pack would have been the result of the gradual decomposition of sodium bicarbonate due to the also gradual pH decrease from 6.2 to 5.7 (Figure 1). The use of hop extract had no effect on the proportion of CO_2_ in the atmosphere. The percentage of O_2_ in the atmosphere was constant during storage. As can be seen in Figure 3, the hop extract reduced the residual O_2_ content in a similar amount regardless of the storage time. This O_2_-consumption effect might be attributed to the action of the polyphenols contained in the hop extract (Hrnčič et al., 2019) that, as part of their antioxidant mechanism, would scavenge O_2_. The oxygen-scavenging ability of polyphenols has been reported previously (Gaikwad and Lee, 2016). The use of hop extract as an ingredient in sausages packed under anaerobic atmosphere would be recommendable to improve their lipid oxidative stability by reducing the presence of residual O_2_.

### 3.2 Changes in volatile compounds

A total of 46 volatile compounds were detected in the headspace of both CON and HOP sausages during the whole storage period. The mean values of the total amount (sum of all) of volatile compounds (expressed as ng of hexanal equivalents) per day or sausage type, and the percentages of the volatile compounds grouped in different chemical families, over the total amount of volatile compounds, are shown in Table 1. The grouping of volatile compounds was carried out following both chemical (functional groups) and potential origin criteria. As explained below, a large part of the volatile compounds identified could be assigned to a single origin, i.e. microbial metabolism, lipid auto-oxidation, or spices and condiments. However, the other part would have had more than one main origin so that its presence and changes are more difficult to explain. The list of individual volatile compounds detected in the sausages and their levels are shown in Table 2. The *P*-levels for the interaction treatment × time were omitted from tables for brevity because they were always >0.1.

**Table 1:** The total sum of volatile compounds (expressed as ng of hexanal equivalents) extracted from the headspace of fresh sausages stored at 2 °C under an anaerobic CO_2_-containing modified atmosphere, and percentage of the different groups of volatiles extracted over the total sum, as a function of storage day (n = 6) and sausage treatment (control sausages, CON, or sausages containing 30 ml/kg of a hop extract, HOP; n = 18).

Volatile group†	Storage day (time)						Treatment (treat)		RMSE	*P*-value	
	0	7	14	21	28	35	CON	HOP		time	treat
Total sum of volatiles (46)	1410.6	813.2	938.7	816.3	1085.8	1174.4	1106.1	972.0	387.9	NS	NS
Abundance of groups (%)											
Acetoin and derivatives (3)	0.2^b^	11.5^ab^	10.7^ab^	18.2^a^	10.6^ab^	11.2^ab^	10.7	10.0	10.0	∗	NS
Branched-chain and aromatic aldehydes (3)	0.1^c^	3.8^c^	6.0^bc^	10.7^ab^	10.5^ab^	12.8^a^	7.2	7.4	5.0	∗∗	NS
Aliphatic esters (2)	n.d.	0.5^c^	1.9^bc^	3.9^ab^	4.1^ab^	4.6^a^	2.8	2.2	1.8	∗∗∗	NS
Aliphatic carbonyls (4)	13.5^ab^	16.1^a^	6.1^bc^	6.0^bc^	3.2^c^	2.8^c^	10.3	5.6	6.3	∗∗	∗
Aliphatic alcohols (4)	6.0	6.4	4.0	6.1	6.7	7.2	7.7	4.4	2.9	NS	∗∗
Aliphatic hydrocarbons (11)	8.3	10.2	17.5	11.8	16.2	12.6	12.4	13.1	10.1	NS	NS
Sulphur compounds (7)	60.1^a^	39.9^b^	44.0^b^	30.0^b^	31.8^b^	31.5^b^	39.1	40.0	10.9	∗∗∗	NS
Terpenes (12)	11.8^ab^	11.7^ab^	9.8^b^	13.4^ab^	17.0^a^	17.3^a^	9.7	17.3	4.3	∗	∗∗∗

n.d.: not detected; under the detection limit (0.1 ng of hexanal equivalents); ^abc^: time-related means in the same row showing different superscripts are significantly different (*P* < 0.05; Duncan's multiple range test); RMSE: root mean square error; *P*-level: NS: not significant; ∗: *P* < 0.05; ∗∗: *P* < 0.01; ∗∗∗: *P* < 0.001.

† Total number of volatiles or number of volatiles in each group in brackets.

**Table 2:** Levels of individual volatile compounds (expressed as ng of hexanal equivalents) extracted from the headspace of fresh sausages stored at 2 °C under anaerobic CO_2_-containing modified atmosphere as a function of storage day (n = 6) and sausage treatment (control sausages, CON, or sausages containing 30 ml/kg of a hop extract, HOP; n = 18).

Compound†	Storage day (time)						Treatment (treat)		RMSE	*P*-value	
	0	7	14	21	28	35	CON	HOP		Time	treat
Acetoin and derivatives											
Diacetyl (600)	3.1^b^	71.1^a^	92.3^a^	96.3^a^	103.7^a^	84.6^a^	77.1	73.2	67.4	∗	NS
Acetoin (740)	n.d.	8.1	9.4	5.8	12.4	13.4	10.8	5.6	11.1	NS	NS
2,3-Butanediol (833)	n.d.	n.d.	n.d.	n.d.	12.0	35.6	4.8	11.1	28.3	NS	NS

Branched-chain and aromatic aldehydes											
Methyl-3-butanal (658)	n.d.	22.7	22.4	13.3	13.8	12.7	18.2	10.1	14.0	NS	NS
Methyl-3-butanol (767)	1.2^c^	4.1^c^	31.0^bc^	52.1^bc^	68.6^ab^	108.7^a^	39.9	48.7	45.1	∗∗	NS
Phenylacetaldehyde (1077)	n.d.	n.d.	0.2^a^	1.0^a^	33.2^b^	34.6^b^	6.0	17.0	40.6	∗	NS

Esters											
Ethylacetate (630)	n.d.	2.74^b^	18.58^ab^	30.86^a^	34.14^a^	39.64^a^	21.97	20.01	19.7	∗∗	NS
Ethylhexanoate (1015)	n.d.	n.d.	0.29^b^	3.43^b^	9.77^a^	10.82^a^	4.31	3.8	4.0	∗∗∗	NS

Aliphatic carbonyls											
Hexanal (811)	176.0^a^	125.7^ab^	52.3^ab^	62.2^bc^	20.1^c^	15.9^c^	108.2	42.5	73.5	∗∗	∗
Heptanal (914)	15.6^a^	4.4^b^	5.0^b^	5.5^b^	1.8^b^	2.6^b^	7.4	4.3	7.0	∗	NS
Nonanal (1118)	7.0	4.7	5.1	12.2	11.7	9.6	8.29	8.45	10.6	NS	NS
2-Heptanone (906)	2.6	1.6	1.8	1.3	1.4	1.8	2.11	1.42	1.7	NS	NS

Aliphatic alcohols											
Pentanol (798)	42.5^a^	20.1^b^	9.6^bc^	11.2^bc^	7.2^bc^	5.3^c^	21.9	10.1	11.2	∗∗∗	∗∗
Hexanol (899)	5.2	6.8	7.7	21.4	26.8	20.5	14.0	15.5	21.0	NS	NS
2-Ethyl-1-hexanol (1051)	2.6^b^	6.5^b^	7.3^b^	9.5^b^	17.6^a^	19.2^a^	14.6	6.3	5.9	∗∗∗	∗∗∗
1-Octen-3-ol (990)	34.7	21.4	14.5	17.4	20.1	33.0	33.0	14.0	23.7	NS	∗

Aliphatic hydrocarbons											
Heptane (700)	0.8	0.6	2.3	3.5	2.8	2.5	2.1	2.1	2.1	NS	NS
Octane (800)	10.8	13.1	18.9	16.7	22.0	20.0	15.7	18.1	10.0	NS	NS
Decane (1000)	13.7	10.9	10.9	19.0	20.3	12.3	14.4	15.4	23.5	NS	NS
Undecane (1100)	1.0	0.5	1.5	3.4	3.4	3.1	2.1	2.0	2.9	NS	NS
Dodecane (1200)	1.0	0.5	0.5	1.1	1.5	1.0	0.8	1.1	1.8	NS	NS
Tridecane (1300)	0.6	0.2	0.4	0.7	1.2	1.3	0.8	0.7	1.2	NS	NS
Tetradecane (1400)	0.3	0.5	1.1	2.1	2.4	3.4	1.5	1.8	2.5	NS	NS
Octadiene (830)	3.6	6.9	5.7	3.7	6.1	2.0	5.8	3.5	5.2	NS	NS
2,2,4,6,6-Pentamethylheptane (991)	80.6	49.0	128.2	79.9	90.4	89.2	95.2	76.3	117.5	NS	NS
Branched alkane (1030)	8.3	4.4	15.6	7.8	9.3	9.1	10.7	7.5	16.1	NS	NS
Branched alkane (1115)	3.7	1.6	4.0	3.3	3.8	5.4	3.9	3.3	7.0	NS	NS

Sulphur compounds											
Allyl mercaptan (600)	94.7	35.8	46.7	12.0	36.2	56.8	47.0	47.0	44.2	NS	NS
Allyl methyl sulphide (706)	599.8^a^	239.2^b^	290.3^b^	160.2^b^	224.4^b^	225.0^b^	302.8	270.2	125.0	∗∗∗	NS
Methyl 1-propenyl sulphide (740)	4.6	1.7	0.7	0.1	0.2	0.6	1.3	1.3	2.8	NS	NS
Dimethyl disulphide (751)	3.8^a^	1.1^ab^	2.4^ab^	1.3^ab^	0.2^b^	n.d	0.6	2.3	2.5	NS	NS
Diallyl sulphide (861)	77.0	20.2	26.8	17.9	36.6	36.8	48.5	22.6	34.2	NS	∗
Methyl allyl disulphide (925)	19.4	7.2	7.5	7.6	9.8	13.9	12.0	9.8	7.9	NS	NS
Diallyl disulphide (1090)	44.3	26.4	18.9	24.6	35.2	32.7	34.5	26.2	27.2	NS	NS

Terpenes											
α-Pinene (938)	11.6^ab^	6.1^c^	7.2^bc^	5.0^c^	11.7^ab^	13.3^a^	8.6	9.6	4.3	∗∗	NS
Camphene (959)	0.9	3.0	1.4	1.2	1.3	1.6	1.4	1.8	2.2	NS	NS
β-Pinene (963)	3.3	2.3	3.8	1.4	2.2	1.7	2.9	2.1	2.7	NS	NS
Sabinene (980)	6.5^ab^	4.8^bc^	2.4^c&^	3.3^bc^	6.3^ab^	8.4^a^	4.7	5.8	2.7	∗∗	NS
Myrcene (998)	31.2	10.4	13.2	12.7	24.2	41.0	5.2	32.6	22.6	NS	∗
Carene (1018)	38.8^abc^	16.3^c^	23.5^bc^	22.2^bc^	43.3^ab^	51.0^a^	31.7	33.3	19.4	∗	NS
Cymene (1032)	3.0	3.0	2.9	2.8	4.6	4.7	3.3	3.7	2.6	NS	NS
Limonene (1038)	47.6	37.1	39.1	54.0	72.4	69.5	42.6	64.0	33.1	NS	NS
Dimethyl styrene (1108)	4.1	5.8	4.8	5.2	7.5	7.6	4.6	7.1	4.3	NS	NS
Copaene (1364)	1.0	0.5	0.2	0.8	2.0	2.2	0.6	1.6	1.3	NS	∗
β-Caryophyllene (1410)	3.3	1.8	2.0	2.4	4.5	6.6	1.6	5.3	3.3	NS	∗∗
Humulene (1465)	4.0	3.4	2.3	3.5	7.5	10.7	0.3	10.1	5.5	NS	∗∗∗

n.d.: not detected, under the detection limit (0.1 ng of hexanal equivalents); ^abc^: time-related means in the same row showing different superscripts are significantly different (*P* < 0.05; Duncan's multiple range test; RMSE: root mean square error; *P*-level: NS: not significant; ∗: *P* < 0.05; ∗∗: *P* < 0.01; ∗∗∗: *P* < 0.001.

† Experimental relative retention index in brackets.

Time had a no significant effect on the total amount of volatile compounds (*P* = 0.078; Table 1), and this amount was not affected using hop extract (*P* = 0.204). Regarding the group percentages, time had a significant effect on the percentages of most of the chemical groups. Acetoin (3-hydroxy-2-butanone) and derivatives (Xiao and Lu, 2014), branched-chain and aromatic aldehydes, and aliphatic esters increased. In contrast, aliphatic carbonyls and sulphur compounds decreased. Significant differences were found between day 0 and days 21 or 28 (the last in the case of aliphatic carbonyls). The use of hop extract affected the aliphatic carbonyl and alcohol percentages negatively, and the terpene percentage positively (Table 1).

As for the individual volatile compounds, some of them were significantly affected by storage time and others by the use of hop extract, with the effect of time being strongest. In general, changes in the volatile compounds detected in the sausage headspace during storage could be explained by molecule degradation or formation reactions, and also to changes in the characteristics of the sausage matrix affecting the release of volatile compounds into the headspace, e.g. the pH changes (Figure 1). Changes in pH modify the capacity of meat proteins to adsorb volatile compounds. It has been reported that a pH change from 6 to 5 in a meat protein solution increases the surface hydrophobicity of myofibrillar proteins. This has been explained by the rebuilding of the protein conformation, and the subsequent changes in protein capacity to absorb volatile compounds (Yang et al., 2017). This capacity has appeared to be dependent not only on the protein conformation change but also on the chemical structure of volatile compounds, i.e. functional group and carbon chain length.

Considering both CON and HOP treatments, the levels of acetoin, diacetyl (2,3-butanedione), and 2,3-butanediol increased during storage (Table 2). Diacetyl, the most abundant, showed a significant increase from day 0–7 (*P* < 0.05). The levels of acetoin could not be quantified until day 7, and those of 2,3-butanediol until day 28, well after the onset of the microbial growth stationary phase (Figure 1). The presence of these compounds in meat has been attributed to microbial use of pyruvate or other intermediary metabolites formed from glucose and amino acid catabolism, the latter occurring under nutritional limiting conditions, such as glucose restriction (Casaburi et al., 2015; Montanari et al., 2018). Thus, in the fresh sausages studied, these volatiles would have been generated as metabolic end-products from sugars at the beginning of the storage and, more importantly, from amino acid catabolism later on, when the sausages supported significant microbial growth. According to Casaburi et al. (2015); Montanari et al. (2018), the presence of those volatile compounds can be related to flavour deterioration and spoilage of meat and fresh meat products during aerobic and anaerobic refrigerated storage.

Taking into account the changes in the microbial population in the sausages described in our previous study (Carballo et al., 2019), the production of acetoin and derivatives could be attributed, to a large degree, to the growth of *Lactobacillus* spp., specifically *L. sakei*. It could also be associated with the growth of *B. thermosphacta* and facultative anaerobic *Enterobacteriaceae*. All those microorganisms are able to generate acetoin from pyruvate (Montanari et al., 2018; Stanborough et al., 2017).

Storage also resulted in a net production of 3-methylbutanal, 3-methylbutanol, and phenylacetaldehyde (Table 2). These are intermediaries from the amino acid catabolism of leucine (the two former) and phenylalanine (the latter) and could have been produced by LAB and *B. thermosphacta* due to their aminotransferase activity (Ardö, 2006; Stanborough et al., 2017). These compounds have been commonly identified in stored meat, where they may play an important role in flavour due to their low odour thresholds, especially that of 3-methylbutanal (Casaburi et al., 2015; Smit et al., 2009). However, this role appears not to have been clearly stated so far.

The other two volatile compounds generated by microorganism metabolism that increased during storage were ethyl acetate and ethyl hexanoate (Table 2). Both have been found to be major esters among those detected in spoiled meat, with the latter having a potentially great impact on odour (Casaburi et al., 2015). It has been suggested that ethyl esters are formed in fermented food by the LAB species via esterification (e.g. from ethanol and acetate) or a transferase reaction, i.e. alcoholysis (from alcohols and acylglycerols), when ethanol is available (Liu et al., 2004). They can also be produced by *Enterobacteriaceae* species and *B. thermosphacta* (Casaburi et al., 2015).

The use of hop extract in the sausage formulation did not affect the levels of any of the above-mentioned volatile compounds related to microbial origin. The scarce effect of hops on the microbial metabolism, in spite of hops being rich in natural antimicrobials, is coherent with the lack of effect of hops on microbial growth in these sausages (Carballo et al., 2019), which was attributed to interactions between hop antimicrobials and the sausage matrix reducing their antimicrobial efficacy.

Most of the straight medium-chain carbonyls and alcohols detected in sausages has been reported to be secondary products from the oxidation of polyunsaturated fatty acids (Meynier et al., 1999). Among them, hexanal appears to be the most abundant in muscle food, and its levels have been proposed as indicators of lipid oxidative status in fresh and cooked meat (Shahidi and Zhong, 2005). Hexanal, heptanal, and pentanol levels showed a steady decrease in the sausages during storage, significantly different for hexanal from day 21, which suggests that lipid oxidation was not relevant during storage under anaerobic atmosphere and low temperatures. The decrease in the levels of those aldehydes can be explained mainly as a result of a negative balance between their formation by lipid oxidation and degradation by reactions with meat components (Shahidi and Zhong, 2005). The levels of hexanal, pentanol, and 1-octen-3-ol were significantly lower in HOP sausages (like the percentages of carbonyls and alcohols). The antioxidant effect of hop extracts (Villalobos-Delgado et al., 2015) was responsible for these reductions.

Apart from lipid auto-oxidation, several aliphatic carbonyls and alcohols, such as hexanal, nonanal, 2-heptanone, hexanol, and 1-octen-3-ol, could also be formed from microbial metabolism during fresh meat aerobic or anaerobic storage, i.e. amino acid catabolism, fatty acid oxidation, or de-hydrogenation of secondary alcohols, with the latter only in the case of 2-heptanone (Casaburi et al., 2015; Ercolini et al., 2009; Pothakos et al., 2015). Individual differences in their microbial and oxidation production rates, and in their reactivity with other sausage compounds, could explain the differences between the patterns followed during sausage storage by the carbonyls and alcohols detected.

The levels of 2-ethylhexanol in sausages showed a steady increase with storage time (significant from day 28). It is an alcohol commonly detected in stored meat that can negatively affect its flavour (Casaburi et al., 2015). To our knowledge, in the literature consulted, its presence in stored meat has not been related to lipid oxidation but to LAB and *B. thermosphacta* metabolism (Casaburi et al., 2015), and especially that of *Carnobacterium maltromaticum* (Ercolini et al., 2009). In agreement, the presence of this species has been detected in the sausages of this study (Carballo et al., 2019). This compound might also have originated from the molecular migration phenomena from the multilayer packaging material (Rivas-Cañedo et al., 2009), probably derived from the plasticiser di(2-ethylhexyl)phthalate (Horn et al., 2004). The levels of 2-ethylhexanol were lower in the HOP sausages. This might be explained as an inhibitory effect of the hop extract on the pathways involved in its production.

Eleven aliphatic hydrocarbons, representing close to 10% of the total volatile compounds, were detected in the sausages without being affected either by storage or hops. Their levels varied highly among samples, and the most abundant was 2,2,4,6,6-pentamethylheptane. Most of the aliphatic hydrocarbons found in the sausages were previously detected in the headspace of meat and fresh meat products (Del Blanco et al., 2017; Rivas-Cañedo et al., 2011). Their origin in meat has been generically associated with lipid oxidation (Madruga et al., 2009) and, more specifically, with migration from packaging (Rivas-Cañedo et al., 2009). The same authors reported the presence of some of the alkanes detected in the present study in the meat, i.e. undecane, tridecane, and 2,2,4,6,6-pentamethylheptane (the most abundant), as the result of migration from the multilayer plastic film used for packaging. The contribution of microorganisms to the formation of hydrocarbons and their impact on flavour seemed to be low (Casaburi et al., 2015).

All of the sulphur-containing volatile compounds detected in this study are major organo-sulphur compounds described in crushed and heated garlic, which are derived from allicin via thiosulphinate degradation (Kim et al., 2011). Five of them, namely allyl mercaptan, allyl methyl sulphide, diallyl sulphide, methyl propenyl disulphide and diallyl disulphide, were the garlic-derived sulphur compounds detected in the headspace of pork patties containing 1.4% or 2.8% fresh garlic as an ingredient (Park and Chin, 2014). As shown in Table 2, the percentage of sulphur compounds and the amount of allyl methyl sulphide (the most abundant) showed a significant decrease from day 0 to day 7. In line with the present results, Park and Chin (2014) also found that garlic-derived sulphur compounds in patty headspace decreased during storage. These experiments were carried out in trays covered by air-permeable film. They attributed part of the decrease to loss of volatile compounds from the patties to the environment. In this study, the packaging material had a reduced gas permeability; thus, the observed decrease might be associated with the loss of sulphur compounds from the sausages to the gas in the bags and to chemical interactions of the sulphur volatiles with compounds in the packaging material or the sausage matrix. All these hypothesised reasons need further studies for validation. The effect of hop extract on sulphur compounds was limited to reduced amounts of diallyl sulphide, which might have interacted with hop components present in the sausages.

Two types of terpenes were found in the sausage headspace: monoterpenes and sesquiterpenes. Their origin could be attributed to the use of black pepper (Jagella and Grosch, 1999) and hop extract (Vázquez-Araújo et al., 2013), which are rich in the terpenes detected. The proportion of terpenes tended to be lower by the middle of storage and higher at the end. This pattern was accentuated in monoterpenes and statistically significant for three, α-pinene, sabinene, and carene. The change in terpene concentration could be attributed, at least partially, to a sausage matrix effect so that more terpenes were released into the headspace at the end of storage as compared with the middle. The lower pH of sausages at the end of storage might have an increased release of terpenes from the sausage to the headspace. Further studies are needed to confirm this hypothesis. Moreover, the hop extract, as expected, contributed significantly to the levels of myrcene, copaene, β-caryophyllene, and humulene, with all of them being reported as the major terpenes in hops (Vázquez-Araújo et al., 2013).

### 3.3 Odour analysis

The scores for spoilage odour intensity are summarised in Table 3. The spoilage score increased during storage for both sausage types. The off-flavour was detected on day 14 and became considerable from day 21 onwards. These patterns were consistent with those of acetic acid and several volatile compounds mainly produced by microbial metabolisms, such as 3-methylbutanol and the ethyl esters, supporting the microbial role in the formation of the spoilage off-odour. Moreover, these odour results suggest a shelf life for both CON and HOP sausages of no more than 14 days, when the spoilage odour was detected. This period is in general agreement with the shelf life of fresh sausages refrigerated under anaerobic atmospheres in other studies (Martínez et al., 2005; Ruiz-Capillas and Jiménez-Colmenero, 2010).

**Table 3:** Spoilage (off-odour) scores (median value obtained from 5-6 panellists per sample) given to fresh lamb sausages stored refrigerated (2 °C) under a 20% CO_2_ and 80% N_2_ atmosphere for up to 35 days as a function of sausage treatment (n = 3; control sausages, CON, or sausages containing 30 ml/kg of a hop extract, HOP).

Treatment	Storage day					
	0	7	14	21	28	35
CON	0	0	0.5	2	2	2
HOP	0	0	1	1.5	2.5	2.5

Spoilage off-odour scale: 0 = none, 1 = slight, 2 = considerable, 3 = extremely perceivable.

## 4 Conclusions

Flavour compounds in fresh sausages packaged under anaerobic CO_2_-containing atmospheres experience complex changes during refrigerated storage as a result of different biochemical reactions that cause odour spoilage. The changes result in a progressive accumulation of a number of volatile compounds considered as end-products of meat spoilage specific microbiota growing under anaerobic and sugar restriction conditions. Among them, acetic acid, 1-methylbutanol, ethyl acetate, and ethyl hexanoate showed levels in sausage headspace directly correlated with storage time. In contrast, lipid oxidation could be not noticeable, i.e. secondary products of lipid oxidation would decrease or maintain their concentration in the sausage headspace during storage. Changes in pH during storage might induce a matrix effect resulting in changes in the release of volatile compounds such as organosulfur compounds or monoterpenes from sausage to the headspace. Further research is needed in this regard.

Hop extract, at the levels used, scarcely interfered with the metabolism of Gram-positive bacteria and appeared not to retard the appearance of sausage odour spoilage. Further research is needed to evaluate the possible effect of using higher amounts of hop extract. In contrast, hop extract at the used amount seemed to reduce the levels of secondary oxidation products in sausages and could exert an oxygen scavenging action. However, the advantage of the use of hop extracts in sausages stored under anaerobic atmospheres would be limited because the formation of lipid auto-oxidation derived volatiles seems not to be the main problem regarding the sausage flavour deterioration. The hop extract also contributed to the terpene content in the sausage headspace, thus potentially affecting sausage flavour.

## Declarations

### Author contribution statement

Diego E. Carballo: Conceived and designed the experiments; Performed the experiments; Analyzed and interpreted the data; Wrote the paper.

Sonia Andrés, Francisco Javier Giráldez: Contributed reagents, materials, analysis tools or data; Wrote the paper.

Ali Khanjari, Sabina Operta: Conceived and designed the experiments; Performed the experiments.

Irma Caro: Conceived and designed the experiments; Performed the experiments; Analyzed and interpreted the data.

Diego Llamazares: Performed the experiments; Analyzed and interpreted the data.

Javier Mateo: Conceived and designed the experiments; Performed the experiments; Analyzed and interpreted the data; Contributed reagents, materials, analysis tools or data; Wrote the paper.

### Funding statement

This work was supported by 10.13039/501100008431Consejería de Educación, Junta de Castilla y León (CSI042 P17). Diego E. Carballo was supported by 10.13039/501100003141Consejo Nacional de Ciencia y Tecnología (CONACYT), México (MEX/Ref. 288189).

### Competing interest statement

The authors declare no conflict of interest.

### Additional information

No additional information is available for this paper.
